# Interpretable Machine Learning Models for Three-Way Classification of Cognitive Workload Levels for Eye-Tracking Features

**DOI:** 10.3390/brainsci11020210

**Published:** 2021-02-09

**Authors:** Monika Kaczorowska, Małgorzata Plechawska-Wójcik, Mikhail Tokovarov

**Affiliations:** Department of Computer Science, Lublin University of Technology, 20-618 Lublin, Poland; m.kaczorowska@pollub.pl (M.K.); m.tokovarov@pollub.pl (M.T.)

**Keywords:** cognitive workload, mutliclass classification, explainable machine learning, eyetracking signal

## Abstract

The paper is focussed on the assessment of cognitive workload level using selected machine learning models. In the study, eye-tracking data were gathered from 29 healthy volunteers during examination with three versions of the computerised version of the digit symbol substitution test (DSST). Understanding cognitive workload is of great importance in analysing human mental fatigue and the performance of intellectual tasks. It is also essential in the context of explanation of the brain cognitive process. Eight three-class classification machine learning models were constructed and analysed. Furthermore, the technique of interpretable machine learning model was applied to obtain the measures of feature importance and its contribution to the brain cognitive functions. The measures allowed improving the quality of classification, simultaneously lowering the number of applied features to six or eight, depending on the model. Moreover, the applied method of explainable machine learning provided valuable insights into understanding the process accompanying various levels of cognitive workload. The main classification performance metrics, such as F1, recall, precision, accuracy, and the area under the Receiver operating characteristic curve (ROC AUC) were used in order to assess the quality of classification quantitatively. The best result obtained on the complete feature set was as high as 0.95 (F1); however, feature importance interpretation allowed increasing the result up to 0.97 with only seven of 20 features applied.

## 1. Introduction

Understanding cognitive workload as a mental effort needed to perform a task [1] is important in human mental fatigue analysis. The diverse complexity of mental tasks requires different levels of concentration. Their understanding and categorising might be useful in the process of modelling information processing capabilities. The level of mental fatigue and its influence on the brain cognitive capability is the subject of numerous research articles [2,3]. Mental fatigue might lead to a decrease of brain cognitive system performance in terms of perception, attention, analysing, and planning [4,5]. What is more, mental fatigue might affect reaction times, target-detection failure, and other objective declines [6].

The literature review conducted shows that the most widely used method of assessment of cognitive workload level in the past employed subjective measures, such as NASA Task Load Index (NASA-TLX) [7,8]. However, the psycho-physiological state might be assessed by objective methods based on bio-signals, such as the eye-tracking technique [9], Galvanic Skin Response (GSR) [10,11], electroencephalogram (EEG), pupillometry, or electrocardiogram (ECG) [12].

Eye-tracking data turn out to be useful in analysing cognitive workload [13]. Benfatto et al. [14] apply eye-tracking features to detect psychological disorders. Workload and performance of skill acquisition based on eye-tracking data are presented in [15]. Eye-movement based classification of visual and linguistic tasks is discussed in [16]. There are also numerous studies presenting cognitive workload classification with a combination of eye-tracking and other bio-signals, such as EEG [17], although the literature does not present numerous cognitive workload classification studies based only on eye-tracking data.

Most cognitive workload classification studies presented in the literature are binary approaches. Among them, the Support Vector Machines (SVM) classifier is one of the most popular [18,19,20]. The above studies, based on different bio-signal data, report its accuracy at the level of even 94–97%. Other popular methods are Linear Discriminant Analysis (LDA) [21], k-Nearest Neighbours (kNN) [22], or Multilayer Perceptron (MLP) [23]. Multiclass problem studies can also be found. Authors applied such methods as SVM ([24] 71%), linear regression ([25] accuracy 82%), or neural networks ([26] 74%, [27] 83%).

Most cognitive workload classification results were achieved in a classical subject-specific approach [27], for which researchers report higher classification performance [20,28]. However, developing a subject-independent classifier allows one to distinguish between cognitive workload levels regardless of external and internal conditions such as the age, time of day, or habits of an examined person. Nevertheless, the literature presents only a few publications with subject-independent approaches [26,27,28]. In [29], the authors conducted a subject-independent and subject-dependent classification based on EEG signals from 14 participants. Thodoroff et al. created a classifier model based on a subject-independent approach [30] using a dataset containing 23 patients.

The aim of the present study includes the following points:

- Performing a multiclass subject-independent classification of cognitive workload levels,
- Examine both classification on the complete feature set and with the application of interpretable machine learning models for feature selection,
- Carrying out a deeper analysis of the features related to the classification of particular levels of cognitive workload.

The dataset used in the study is eye-tracking and user performance data gathered from 29 participants while solving a computerised version of the digit symbol substitution test (DSST).

The digit symbol substitution test (DSST) [31] is a cognitive tool introduced as a paper-and-pencil test originally applied in order to understand human associative learning. Currently, this test is commonly applied in clinical neuropsychology to measure cognitive dysfunction and is often present in cognitive and neuropsychological test batteries [32,33]. The DSST allows one to check the patient’s processing speed, memory, and executive functioning [34].

The rest of the paper is structured as follows. The review of the literature is presented in Section 2, while the research procedures covering the computer application, equipment, and experiment details are discussed in Section 3. Section 4 presents the methods applied in data processing, classification, and statistical analysis procedures. Results are discussed in Section 5. Section 6 contains an analysis and discussion of the extracted feature importance measures. Section 7 concludes the paper.

## 2. Related Work

Table 1 shows a review of the literature where the numbers of participants and cognitive workload levels are presented. The approach column indicates a subject-dependent (sd) or subject-independent (si) approach of classification. For the scientific articles, the results of classification are presented as well. In [35], the authors wrote about labelling cognitive workload data using three methods: difficulty split by expert, the Rash model, and the stress–strain model. The most common method is based on task difficulty conditions named difficulty split labelling, while the Rash and the stress–strain models allow adjusting the cognitive workload level to each participant separately. The authors conducted the examination on 34 participants, obtaining data to label and classify the level of cognitive workload [35]. Lobo et al. published a study on the classifying levels of cognitive workload based on eye-tracker and EEG data [17]. They conducted a three-class classification where they created three levels of cognitive workload and applied a subject-independent approach based on 21 observations. Both the experts and novices took part in the experiment [36], having a low level of cognitive workload while the novices had a higher one [36]. The authors conducted a two-class subject-independent classification based on data from 14 participants. In [37], the authors carried out the experiment asking 35 participants to play two games with different levels of difficulty. 

Almogbel et al. published a study examining the levels of cognitive workload in the process of playing a computer game [38]. The data were collected from one participant, and a classification of low and high level of cognitive workload was obtained. The same authors published another paper where they tried to classify three and six levels of cognitive workload on the basis of data from one participant in the process of playing a computer game [39]. In [38,39], the authors applied a subject-dependent approach to create a classification model. Study [40] attempted to classify low and high levels of cognitive workload defined on the basis of data gathered from eight participants. In [41], a subject-independent classifier was made on the basis of data from 12 participants. The experiment was conducted using arithmetical tasks defining seven levels of cognitive workload, where the first level was the easiest and contained one and two-digit numbers, and the seventh level was related to arithmetical tasks on three-digit numbers with three carries. The authors attempted to classify the three and two cognitive workload levels using a subject-independent and dependent approach based on the pupil data from 25 participants [42]. In [43], two approaches were applied to create a classification model as well. The authors considered the prediction of a driver’s cognitive workload: good or poor driving performance state while driving based on EEG data from 37 participants. In [44], the authors applied eye tracking in virtual reality (VR) and augmented reality (AR) technology to classify the cognitive load of drivers under critical situations. Two-class subject-independent classification was conducted using several types of classifiers: SVM, decision tree, random forest, and k-Nearest Neighbours and used five metrics: accuracy, precision, recall, and F1-score. Data were gathered from 16 participants and two levels of cognitive load were defined: low and high. 

Fridman and colleagues [45] conducted the experiment based on making videos of real-time cognitive load in various contexts which were corresponding to cognitive load level. A total of 92 participants took part in the experiment, and three-level classification was applied using convolutional deep neural network 3D and Hidden Markov Model. In [45], a subject-independent approach was used to create the classification model based on eye images extracted from videos. In [46], the authors noticed that most of the previous research was based on data not related to older adults. They mentioned that the changes in eye-tracking could appear in older adults, so they conducted an experiment where older adults were asked to watch the video clips. A two-level classification model was created on the basis of the data gathered from 12 participants. The model is able to classify the fatigue and non-fatigue state independently from the participant’s age. In [47], the authors presented an interesting approach to cognitive workload estimation using EEG signals with the application of a deep convolutional neural network with residual connections and a gated recurrent unit (GRU). A high accuracy of subject-independent classification was reported for an approach with four workload levels. In [48], a model capable of distinguishing between two cognitive workload levels was developed. The authors proposed a novel approach consisting of two steps that included the initial training of a set of participant-specific classifiers and then combining the trained classifiers to address the problem of subject-independent cognitive workload estimation. In [49], Custom Domain Adaptation (CDA) was used to develop a highly efficient classifier, which was trained with the same dataset as it was applied in [47].

## 3. The Research Procedure

### 3.1. The Computer Application

**Figure 1:**
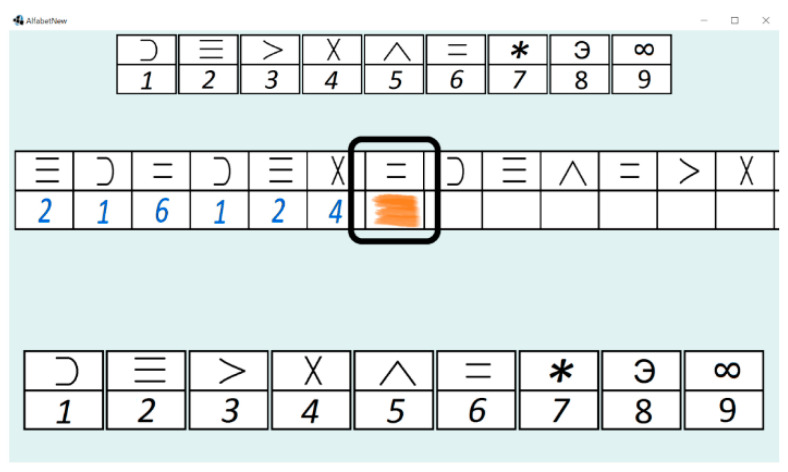
The interface of the application. The highlighted area presents the current symbol to be matched.

In the present study, the computerised version of the DSST test [50,51] was applied. A subject was asked to match symbols to numbers according to a key located at the bottom of the screen. To assign a particular symbol, a subject had to click on the key corresponding to this symbol. A graphical frame (Figure 1) marked the currently active letter, and after assigning a symbol, the frame moved to the next letter. The subject matched symbols to subsequent letters within the specified time. Subsequent letters were generated randomly and with repetition, and they appeared continuously within the defined time.

The number of symbols and the time is defined in the application settings. In the case study, three DSST parts were applied:

- Part 1—low-level cognitive workload: four different symbols to choose from, 90 s test length;
- Part 2—medium-level cognitive workload: nine different symbols to choose from, 90 s test length;
- Part 3—hard-level cognitive workload: nine different symbols to choose from, 180 s test length.

These three parts correspond to the classes of cognitive workload, defined in Section 3.3. Three levels of cognitive workload were empirically defined and separated on the basis of a preliminary pilotage study performed on a small group of two participants with a profile consistent with the characteristics of the study participants (they did not participate in the target study). The number of symbols and the duration of the test were set in an interview carried out after this preliminary examination.

The Java 8.0 programming language was the main tool used for developing the application; it was designed to be operated by a computer mouse. The interface of the computerised version is presented in Figure 1. The legend containing the characters used and the digits assigned to them is shown at the top in the form of the table. The user can select a specific character by clicking the proper cell in the table at the bottom of the window. The central part contains the currently active task (a user is supposed to click the character with digit 6). The left part of the central table presents the history of the tasks, and the right part shows the upcoming tasks.

### 3.2. Setup and Equipment

The described experiment was carried out in a laboratory with standard fluorescent light. In order to ensure stable, equal conditions for all participants, the outside light was blocked. The activity of eyes was registered by a Tobii Pro TX300 screen-based eye tracker (Tobii AB, Stockholm, Sweden) utilising near-infrared technology [52]. The video-oculography method in the said device is based on corneal reflection as well as the dark pupil method (VOG). It collects the data related to binocular gaze with the frequency of 300 Hz for studies of saccades, fixations, and blinks. The sampling rate variability is less than 0.3%. The gaze precision (binocular) is 0.07°, and the gaze accuracy is 0.4°. The eye tracker is built into a monitor (23″ TFT monitor at 60 Hz) connected to the computer (laptop computer Asus G750JX with 8 GB of RAM and processor Intel Core i7–4700HQ) on which the experiment was carried out. Tracking is proceeded for each eye separately.

The experiment was designed in Tobii Studio 3.2, which is the software compatible with the Tobii Pro TX300 eye tracker, and it is dedicated to preparing and analysing eye-tracking tests. Visual stimuli were presented on a separate monitor (23″ TFT monitor with 60 Hz of refresh rate). During the experiment, the participants occupied a seated position in such a way that the screen was from 50 to 80 cm away from the participant depending on the fact of which position was convenient for the participant to work with the computer. The experiment was preceded by the nine-point calibration procedure realised for each participant, so the distance from the monitor within the mentioned range did not affect the results of the experiment. The calibration procedure is performed separately for each eye. Identical hardware and software settings were used for examining all the participants.

The Tobii Studio was applied to export eye activities gathered during the experiment. The signal lost was used to check the tracking ratio to ensure the proper quality of data. Eye activities exported from the experiments were related to several measures:

- Fixations [53], originally defined as the period of uptake of visual information, when a participant holds eyes relatively stable in a particular position. Single fixation occurs between two consecutive saccades. All visual input occurs [54] during fixations.
- Saccades [53] are defined as the rapid eye movement occurring between fixations. During saccades, the eye gaze is moved from one point to another to bring the part of visual information onto the most sensitive part of the retina in order to retrieve information [54].
- Blinks derived as zero data embedded in two saccadic events.
- The blink was identified as zero data is embedded in two saccadic events [46,55,56].
- Pupillary response understood as pupil size. The Tobii Studio applies the dark pupil eye-tracking method.

Fixations and saccades are detected in the Tobii Studio with the Velocity–Threshold Identification (I-VT) fixation classification algorithm [57], which classifies the eye movements using directional velocity shifts of the eye. The velocity threshold parameter was set to 30°/s [58]. This is the recommended value sufficient for recording with the average level of noise. The research on the I-VT velocity threshold parameter for the Tobii TX300 eye-tracker show that it provides stability when the I-VT threshold is between 20°/s and 40°/s [52]. The amplitude of saccades and blinks were determined directly from the eye tracker via 3D eye position and screen gaze points.

### 3.3. Experiment

The experiment was applied on a group of 29 participants aged 20 to 24 (mean = 20.61 years, std. dev. = 1.54). The tested group was homogeneous. Healthy participants (23 males, six females) were recruited among second- and third-year students of the BSc degree in computer science. The participants had normal/corrected to normal vision. The acceptable level of data activity recorded by the eye-tracker was set to 90%. Originally, 30 participants were invited to participate in the study; however, one participant was discarded due to poor data eye-tracking quality caused by excessive body and head movement (the data activity level recorded by the eye-tracker for this participant was 70%).

The research was approved by the Committee of the Lublin University of Technology (Approval ID 2/2018 from 19 October 2018). The participation was voluntary, and all participants received information about the study.

**Figure 2:**
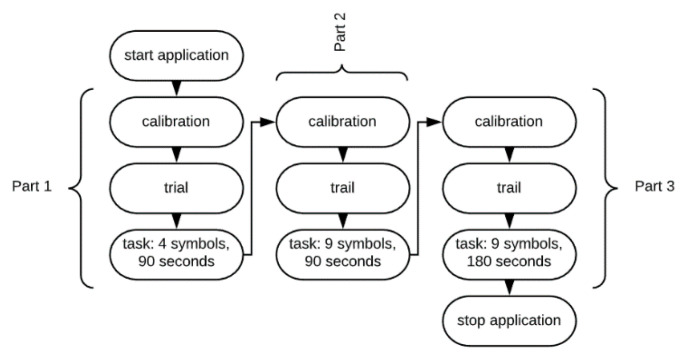
The procedure of the experiment.

A single participant was examined for approximately 15 min. The experiment was divided into three parts. Each part was preceded by a calibration phase. The calibration phase was run on the eye-tracker as a built-in procedure. The next step contained the instructions demonstrated to the participants on the screen. The participants were asked to create as many matches as possible by assigning symbols to the appearing letters. The assigning was done by clicking on a particular symbol on the key located at the bottom of the screen (see Figure 1). Afterwards, the participants went through the three parts of the DSST using the computer application. Every experiment stage contained a different number of symbols to assign and lasted a different period of time. Each stage started from a short trial including nine symbols, and the participants were supposed to familiarise with the task by performing the trial. After finishing the initial part, a participant could start the main part of the test stage. Figure 2 presents the procedure of the experiment.

### 3.4. Dataset

The dataset consists of files generated from the eye-tracker and files generated from the application. The files generated from the eye-tracker consist of the time stamps and the data that are related to such eye activity as fixations, saccades, blinks, etc. The files generated by application include the time stamps and the data that are related to DSST test results such as number of errors or number of responses. The total number of generated files per participant equals six, i.e., three files from the eye-tracker and three files from the application. The data from each task were saved in a separate file.

Eye activity-related and DSST test results-related data exported from the eye tracker covering 20 features are presented below:

- Fixation-related features: fixation number (total number of fixations), mean duration of fixation, standard deviation of fixation duration, maximum fixation duration, minimum fixation duration.
- Saccade-related features: saccade number (total number of saccades), mean duration of saccades, mean amplitude of saccades, standard deviation of saccades amplitude, maximum saccade amplitude, minimum saccade amplitude.
- Blink-related features: blink number (total number of blinks), mean of blink duration.
- Pupillary response features: mean of left pupil diameter, mean of right pupil diameter, standard deviation of left pupil diameter, standard deviation of right pupil diameter.
- DSST test results-related features: number of errors (total number of errors), mean response time, response number (total number of responses).

The listed eye-activity related features were chosen as the most informative ones available in the eye tracker software. Features related to fixations and saccades are the most common eye-tracking features analysed in the literature [54] in such areas as psychology and neuroscience, including behavioural patterns, mental fatigue, and disorders analysis [46,57]. Although in the literature the most common approaches consider only the main fixation- and saccade-related features such as the total number of saccades, mean duration of saccades, total number of blinks, and mean of blink duration, we decided to consider also an additional set of features including standard deviation of saccades amplitude, standard deviation of fixation duration, maximum and minimum saccade amplitude, and fixation duration. Even though these features are not widely applied in cognitive workload research, they were included in the analysis to check its possible usefulness in the process of classification. Standard deviation, mean and skewness of fixation duration, and saccade amplitude have been previously applied in the task of classifying mental states [59]. Standard deviations of the saccade parameters were also used in the analysis of eye-movement relation to the age of the participants [60]. Distributions of fixation durations and of saccade amplitudes were analysed also in the context of classifying eye fixations [61]. Motivation to analyse maximum and minimum values (included in the analysis after correction of outliers) as well as standard deviation of fixation duration and saccade amplitude (as a measure of variance or dispersion) was related to potential statistical differences between particular cognitive workload levels.

Blink-related features as well as pupillary response features were proved to indicate the dynamics of the cognitive process [1,62,63]. Pupil diameter-related features were measured separately for each eye and needed additional preprocessing steps. The pupil size was reported in millimeters, and it was estimated based on the magnification effect achieved by both the spherical cornea and the distance to the eye [46]. Linear interpolation was applied in order to reduce the impact of blinking or artifacts [64]. A sudden pupil size change of 0.1 mm, within a 3 ms time span, was marked as an artifact [64,65]. Missing data, especially data related to blinks, were ignored and were not included in the further processing. A subtractive baseline correction (corrected pupil size = pupil size − baseline) [66] has been applied to the pupil size data. The baseline has been estimated based on 100 ms fragment recorded before the main experimental procedure (during the welcome page displayed). The DSST test results-related features were included to complete the analysis process.

## 4. Methods Applied

### 4.1. Data Processing

Eye activity data and DSST test results were analysed off-line using custom programs written in MATLAB 2020a and Python 3.6. The procedure of data processing was divided into 6 steps:

- Data acquisition
- Data synchronisation
- Feature extraction
- Feature normalisation
- Feature selection
- Training and testing classification models.

Six files were generated per participant, three for both parts: one file from the computer application and one from the eye-tracker. The essential part of preprocessing was the procedure of synchronisation, which was necessary to prepare the dataset. It allowed combining the files from the eye-tracker and application and obtaining one file per each part of the experiment. Every participant provided three observations: the first one corresponded to a low level of cognitive workload (file 1), the second one was labelled as moderate (file 2), and the last one had the signal associated with a high level of cognitive workload (file 3). The dataset included 87 observations in total (3 observations per participant). Every observation consisted of independent features and the class label (low, moderate, or high). The values of independent features were obtained by means of a feature extraction step, leading to the twenty features listed in Section 3.4. The feature normalisation step was the next stage in the procedure of data processing; it was necessary to ensure a uniform scale of the features.

### 4.2. Feature Selection

In order to obtain the values of feature importance measure, the logistic regression model was used. Due to its properties, logistic regression is commonly used in the applications where deep understanding of the reasons behind the decision taken by the model is necessary [67]. The model of logistic regression can be expressed by the formulae below. First, the weighted sum of the feature values is computed as shown below:

(1) $$S=\sum_{i=1}^{n}w_{i}\;\cdot \;a_{i}+w_{0}.$$

The result is calculated with the use of the following logistic function:

(2) $$p=\frac{1}{1+e^{-s}}$$

where

**Figure 3:**
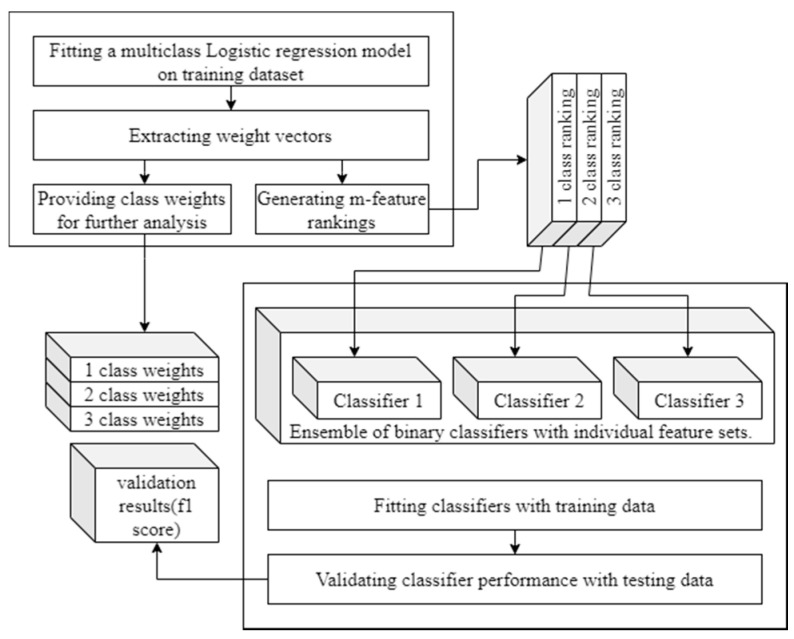
Feature selection experiment flowchart.

- p is the probability of the fact that the classified sample belongs to the positive class,
- i is the order number of the feature,
- $w_{i}$, (i∈ [1,n]) is the weight of the i-th feature; the lower the absolute value, the less important the feature, and conversely, higher absolute values of the weights correspond to the features producing great influence on the model’s decisions,
- $w_{0}$ is the bias,

$a_{i}$, (i∈ [1,n]) is the value of i-th feature.The values of weights {$w_{i}$}, representing feature importance, are obtained in the process of model training. Hence, training a logistic regression model on the complete feature set is a starting point for feature selection. Figure 3 presents the process of feature selection and appliance of its results in classification. The logistic regression models applied for feature selection include also regularisation elements; after initial consideration, elastic net, being the linear combination of L1 and L2 regularisation, was applied. The approach allowed mitigating possible correlations between features, so that in a pair of correlated features, one obtained a higher importance weight and the other was assigned a notably lower weight.

### 4.3. Classification

The aim of the classification process was to assign observations into one of the three classes:

- Class 1—observations with low level of cognitive workload
- Class 2—observations with moderate level of cognitive workload
- Class 3—observations with high level of cognitive workload

Classes correspond to three cognitive workload levels applied in the DSST application.

Various classification methods, such as SVM, kNN, Random Forest, Multilayer Perceptron (MLP), and Logistic Regression were applied in order to produce initial results, showing the general perspectives of eye-tracking data as the source of information for cognitive workload assessment.

The classifier models mentioned had the following hyperparameters:

- kNN—nearest neighbour number: 5
- Random Forest—tree number: 100
- MLP—two hidden layers: 32 and 16 neurons; optimiser: Adam; learning rate: 0.0001; activation function: relu (rectifier linear unit)

Afterwards, logistic regression was used for extracting feature weights representing their importance in the process of distinguishing between particular levels of cognitive workload. The influence of selected features was tested for the classifiers mentioned before.

The mentioned classification models were chosen due to the following reasons: lower number of parameters (compared to deep learning models), lower tendency to overfit on small datasets (compared to deep learning models), low computational cost allowing running multiple experiments in a reasonable amount of time. Moreover, selection of algorithm was performed considering the other studies in the field of cognitive workload classification.

The dataset was shuffled in random order and divided into train and test datasets. Data from every participant could be used only in one dataset: train or test, in order to ensure a truly subject-independent approach, so that the signals of the test dataset persons would be completely unknown for the model. Approximately 20% of the input dataset, which corresponds to 6 participants, was used for testing.

A range of classical machine learning models were used. The applied approach included the initial state, where the classifiers were tested on the complete feature set and afterwards the main analysis where the feature selection was conducted. This solution allows optimising calculation, avoiding the models that provide worse results at the very beginning.

### 4.4. Statistical Analysis

In order to compare the variance of values (for 20 features) from three DSST parts, one-way ANOVA analysis was used. The Kolmogorov–Smirnov test (K-S test) and the Levene test were used to test the assumptions of the ANOVA analysis. The K-S test was performed to verify that variables had a normal distribution, the Levene test was used to check that the variance of the data from three parts of the DSST test was equal. Finally, the Tukey’s honest significant difference test (Tukey’s HSD) post-hoc test was performed to identify which pairs of the DSST test parts had statistically significant differences. The analysis was carried out in MATLAB 2020a software using Statistical and Machine Learning Toolbox.

## 5. Results

### 5.1. Classification Results

The three-class subject-independent classification was conducted and the F1 score of a selected classifier for a complete feature set is presented in Table 1. The following classifiers were applied: SVM with linear, quadratic, and cubic kernels, Logistic Regression, Decision Tree, kNN, Multilayer Perceptron, and Random Forest. The learning process including a train–test cycle was repeated 200 times in order to ensure the stability of results (the number was obtained empirically). Train and test datasets were formed randomly for every repetition. In order to ensure the methodological correctness of the experiments, the procedure of feature selection was performed on the training set independently in every repetition. Table 2 and Table 3 present the results of numerical experiments: the main metrics allowing to assess the quality of classification: recall, precision, F1, accuracy, and ROC AUC.

All the measures, except for accuracy, were computed in a multiclass way; i.e., first, a measure was computed for the separate classes, and the final value was obtained as the mean of class-wise measure values with the weights proportional to the content of specific class samples in the test set.

Table 2 shows the values of classification performance measures obtained for the complete feature set; Table 3 presents the results obtained with the use of the feature selection procedure, based on elastic net, which is composed of logistic regression with linearly combined L1 and L2 regularization.

**Figure 4:**
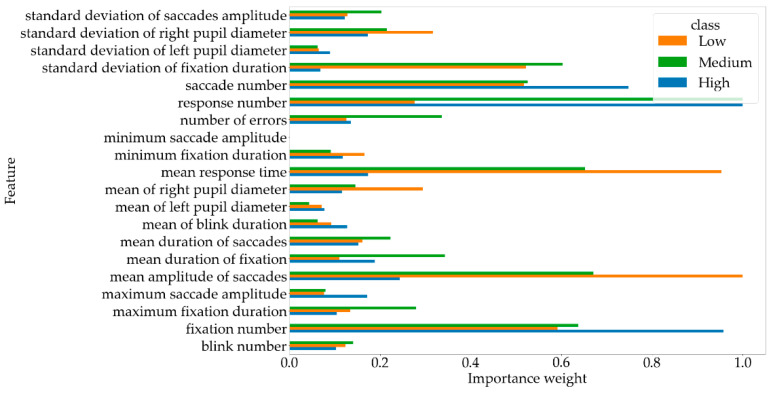
Separate feature importance weights.

As it may be noticed, the best classification quality was achieved by MLP, SVM with linear kernel, and Logistic Regression. The mentioned models required correspondingly five, five, and four features per class. So, the total number of features was as high as seven, seven, and six—the numbers are obtained as the number of common features in the first five and four rows of Table 4, presenting the features ranked with respect to their importance for classifying a sample as an instance of the particular classes. Figure 4 presents the weights of the specific features for particular classes, which were marked with the color and shape of the markers. The whiskers represent the standard deviation of the values. Figure 4 presents the weights of the specific features, so, in addition to the rankings, the importance measures can be compared as well, so one can observe not only which features are more important but also quantitatively examine the difference of importance measures; e.g., it can be noticed that some features are significantly more important: mean amplitude of saccades, number of fixations, response number as well as mean response time.

**Figure 5:**
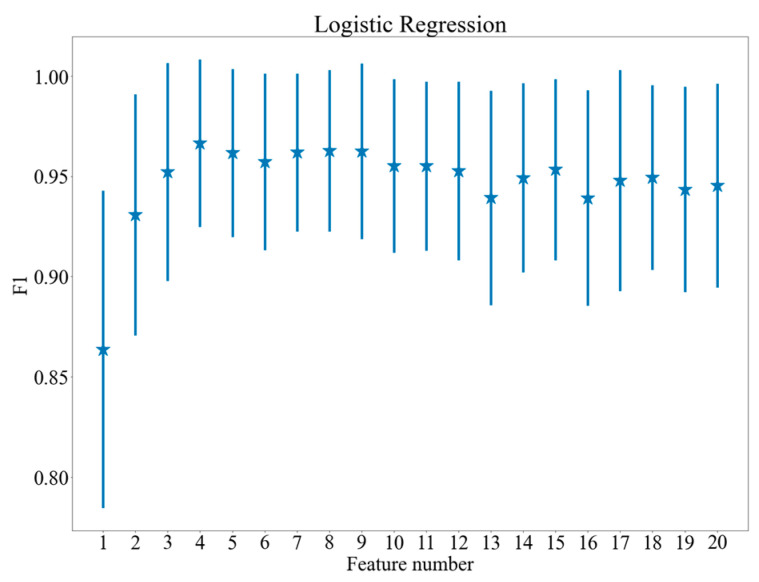
F1 scores for various feature numbers. Logistic Regression.

**Figure 6:**
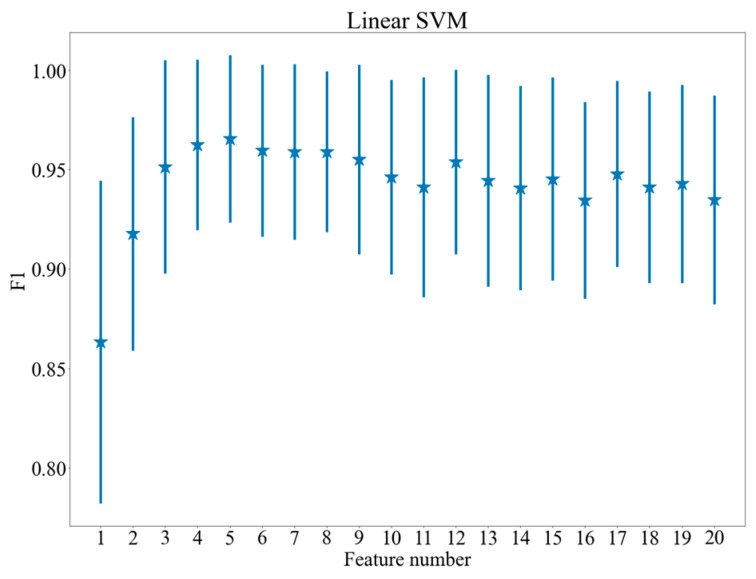
F1 scores for various feature numbers. Linear Support Vector Machines (Linear SVM).

**Figure 7:**
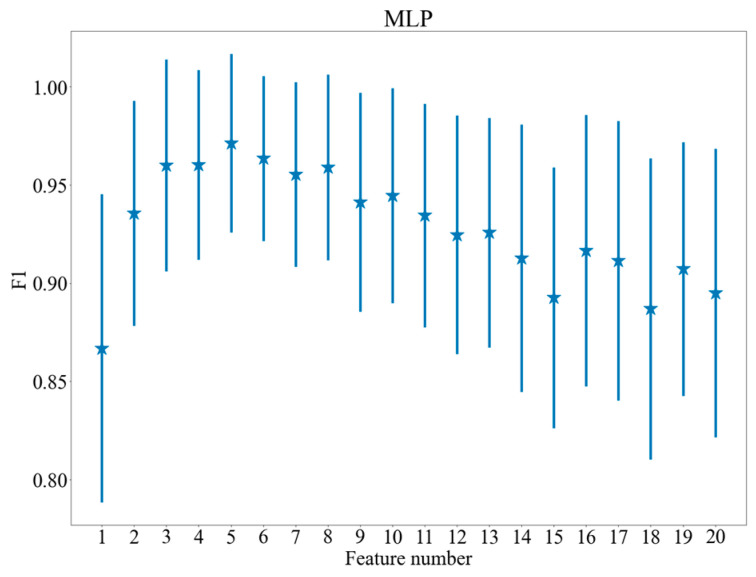
F1 scores for various feature numbers. Multilayer Perceptron (MLP).

Figure 5, Figure 6 and Figure 7 show the dependence between the number of features applied and the results of the models, which achieved the highest values of F1 score after feature selection. The vertical lines indicate standard deviation of the results. As may be observed in the figures, the procedure of feature selection influences classification performance positively: all the plots presented in Figure 5, Figure 6 and Figure 7 demonstrate a distinctive maximum for specific feature number and decrease of the F1 measure for a higher feature number (for SVM and logistic regression). Figure 5, Figure 6 and Figure 7 present quantitative evidence of positive effect produced by feature selection and demonstrate that together with the decrease of computing complexity, an improvement in classification quality is achieved by selecting proper features.

Table 5, Table 6 and Table 7 present the mean confusion matrices of the models that provided the best performance, namely: SVM with a linear kernel, logistic regression, MLP. For example, for the SVM classifier, the observations from class 1 and class 2 are more similar to each other than the observations from class 3. This can be seen in the mean confusion matrix. All the observations of class 3 from the test dataset were classified correctly. On average, 5.62 observations of the first class were classified in the proper way and 0.38 observations from the first class were classified as second-class observations. The mean of 5.77 observation from the second class were classified in a proper way, and 0.23 observations from the second class were classified as first class. Only 0.005 observations on average were classified as the observations of the second class being the observations of the third class, while no observations of the third class were classified as first class.

### 5.2. Statistical Results

The K-S test revealed three features with non-normal distribution (minimum fixation duration, minimum saccade amplitude, and number of blinks). The Levene test found five features with unequal variances (number of fixations, minimum fixation duration, number of saccades, minimum saccade amplitude, number of blinks). As a result, the ANOVA analysis was performed for 15 features. It found seven features for which differences between their mean values were significant (*p*-value 0.05). The Tukey’s HSD post-hoc test identified pairs of DSST parts which presented statistically significant differences.

Table 8 presents significant results (*p*-value < 0.05) of the ANOVA analysis and a post hoc test for selected features. Significant differences were observed mainly between class 1 and class 2, as well as between class 2 and class 3. Only one feature, maximum saccade amplitude, presented significant differences between class 2 and class 3 (*p*-value = 0.046).

**Figure 8:**
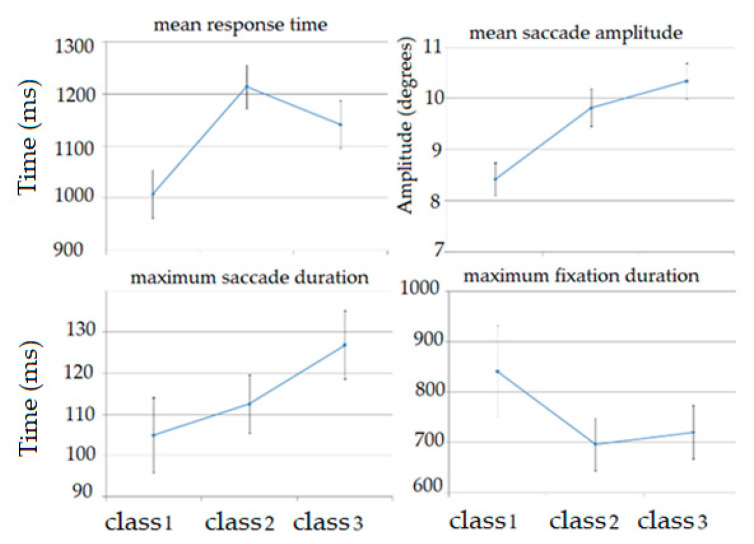
The comparison of mean values from the three-part digit symbol substitution test (DSST) for selected features.

Figure 8 shows the comparison of mean values of the features that demonstrated the significant differences for various classes of cognitive workload.

### 5.3. Cognitive Factors Analysis

The topic of cognitive workload assessment also requires cognitive factor analysis. The term cognitive factors refers to characteristics of the person that affect his/her performance and learning effectiveness. Cognitive factors include such functions as memory, reasoning, and attention. Analysis of eye activity as well as DSST features allows estimating the cognitive factors. The procedure of feature selection run with the use of logistic regression model enabled obtaining the most valuable features, showing that the most important feature subsets are fixation and saccade-related features. Those kinds of features correspond to the intensity of eye movement, which shows a higher degree of attention during the performance of more complicated tasks, which is demonstrated especially well in Figure 8: in maximum fixation duration, we see that with the increase of task level from low to medium, the maximal duration of fixation decreases, which shows that the gaze of the examined person on average stays shorter in one position, which can be more evidence of higher attention in these tasks; the rest of the features presented in Figure 8 also support this thesis.

## 6. Discussion

The aim of the study was to perform the multiclass subject-independent classification of cognitive workload level with both interpretable and noninterpretable machine learning models. A three-class subject-independent classification was performed on the basis of the dataset containing eye-tracking and user performance data. The study assessed mental fatigue with features based on eye-tracking and DSST test results. The data were gathered in a case study of three versions of the computerised Digit Symbol Substitution Test (DSST). An interpretable machine learning model was used for selecting the most valuable features, which allowed improving the result of classification and obtaining insights that enabled understanding the process of mental fatigue.

Eight machine learning models were built and compared. It is a common approach to run an initial stage of classification tests with the use of multiple classifier models in order to find the most promising ones for further analysis. The aim of our work was not only to obtain the highest possible result of the classification but also to gain the feature interpretability especially in the case of subject-independent classification. The initial stage was provided by SVM with linear, quadratic, and cubic kernel, Logistic Regression, kNN, Decision Tree, MLP, and Random Forest. The learning process for each model was repeated 200 times.

Logistic Regression, chosen in the study as a feature selection tool, is commonly applied as an interpretable machine learning model, as it assigns specific weights to separate features, which allows assessing their importance quantitatively and hence creating a ranking. Logistic Regression was applied for feature selection, as it is commonly used in the cases requiring interpretable machine learning. It allowed improving the result from 0.95 to 0.97 (SVM with linear kernel) using only five features per binary classifier out of 20. Logistic Regression demonstrated a similar result with an even lower feature number (4). MLP also ensured high performance, which required five features. Based on the obtained results, it may be noticed that the most reasonable solution is to use an SVM/logistic regression model in the present problem.

The DSST test used in the study was a computerised version of the classical paper-and-pencil test. This cognitive test was chosen in the study as it was sensitive to changes in cognitive functioning, and its performance correlates with the ability to accomplish everyday tasks. Although originally, the test was designed to measure cognitive dysfunction, we decided to apply it in the study, as it is easy to use and it engages the memory and concentration of the participant. A preliminary pilot study was applied in order to define the number of symbols and duration of the test. The original version of the test assumes nine different symbols, which were generated in a random and repeated way to be assigned over a 90 s period. We decided to use this setting for the medium level of cognitive workload, whereas the settings for the low and hard level of cognitive workload were defined in an interview with the pilot study participants. However, the limitation of such an approach is that the level of cognitive workload is set permanently for all participants independently of their abilities and IQ level. It is worth noting that there are some methods dedicated for the evaluation of mental fatigue of particular participants. These methods take advantage of the capacity models (e.g., the Rasch and strain–stress model) and allow adjusting the cognitive workload assessment to a specific person, taking into consideration his/her mental abilities. On the other hand, these methods are based on surveys (e.g., NASA-TLX scale) and gather subjective assessment of the participant, which might also be blurred with different factors. Moreover, there are many various aspects affecting the subjective cognitive workload assessment, which are hard to consider and explain. A problem that can appear here is a more complicated structure of the experiment due to a possible lack of balance between the classes in this case. Nevertheless, supplementing the study with a mental fatigue evaluation of each participant might provide new insights into the analysis results and will be performed as a future work.

The paper presents the classification process based on eye activity gathered with the eye-tracking technique. This dataset is supported with features retrieved directly from the DSST application. The study includes general eye activity features related to visual fixations and saccadic movements, blinking, and pupil characteristics. Information about location indicating where participants were looking was not included. Such data might be used to map eye position onto the visual objects displayed on the screen; however, it is doubtful whether these results could increase the accuracy of the classification. On the other hand, such data could provide information about the test results, although these results are obtained directly from the DSST application.

Classification results and the feature ranking obtained in the study strongly suggest that the performed tasks have a systematic influence on eye movements. The results prove the relation between the participants’ engagement in the task combined with their cognitive state and their eye activity. The results give insights into understanding the dependence between eye movements and cognitive factors. However, further research is necessary to explain these dependences among different participant groups and different stimulus types. Although the results of our study suggest that eye movement-related features might be applied in the process of cognitive state assessment, there is a possibility that the type of tasks, graphical interface, or even initial mental state of the participant might affect the results. However, both sets of features applied in the study (eye movement-related features and DSST test-related features) are objective measures independent of the subjective assessment of individual participants. What is more, the eye tracking-based features chosen in the study are a natural type of response gathered from a non-invasive source and obtained without any training or additional activity. A limitation of the work is the relatively small number of participants. Even if the group of 29 participants turned out to be sufficient for performing the statistical analysis, further studies are definitely necessary to confirm the results over a broader group of participants. What is more, we also plan to check the influence of the task order by randomising it in the experiment.

The highest F1 rate was achieved for SVM with a linear kernel after performing feature selection. Three binary classification models were used for distinguishing between three classes, each of them used an individual six-feature set obtained from the ranking built in the stage of feature selection (the rankings are presented in Table 4). The union of the binary model feature sets contains seven features: mean saccade amplitude, mean response time, fixation number, standard deviation of fixation duration, saccade number, response number, and mean duration of fixation. It might be noticed that the majority of the selected features are related to fixation (standard deviation of fixation duration, fixation number, mean duration of fixation) and saccadic eye movements (mean saccade amplitude, saccade number). It worth noting that only a standard deviation of fixation duration was important among the group containing an additional set of features (including standard deviation of saccades amplitude and fixation duration as well as maximum and minimum saccade amplitude and fixation duration). Such phenomenon could be related to the high variability of fixation duration among particular cognitive workload levels caused by possible physical eye fatigue, although this issue needs further investigation. No blink or pupillarity-related features were included in the set of seven selected features. This result suggests that the most commonly used eye-tracking features related to fixation and saccades are the most informative. The obtained results show that the cognitive workload level is related to the number of saccadic movements and fixation duration. Surprisingly, the analysis has shown low importance of blink-related features, which happened to be low discriminative in terms of cognitive workload. Additionally, according to previous expectations, the features related to DSST results also proved to be important for distinguishing between cognitive workload levels, which is quite intuitive; i.e., a more complicated task requires more time to solve and the probability of error is higher. The complete feature rankings are presented in Table 4. The feature rankings can be analytically understood and interpreted; for example, the analysis revealed that the response number was the most important feature for distinguishing the high level of cognitive workload, which is quite intuitive, as complicated tasks require a longer time to solve. More valuable information was related to the next features: fixation number and saccade number, which is also intuitive: participants tend to move their gaze more rapidly during solving more complex tasks. The results presented in Figure 5 allow comparing the scale of separate feature importance values, showing that some features are significantly more important than others, e.g., mean saccade amplitude, which shows that while solving easier tasks, the participants could move their gaze wider, producing longer saccades.

It has to be taken into account that differences in education, performed job, experience, and age can cause complication in the analysis, and results might differ between these groups. Thus, due to the approximate mental homogeneity of the examined group in the present research, the results show the relation between cognitive workload level and eye activity especially adapted to analytical minds of students of technical specialties.

The statistical analysis was based on the ANOVA procedure. Statistically significant differences for all classes were revealed for the maximum saccade amplitude feature, but the difference was observed only in the following pairs: class 1 and class 3, class 2 and class 3. The most significant differences were observed between class 1, which corresponds to a low cognitive workload level, and class 3, which is related to a high cognitive workload level, as they are the farthest from each other. However, the differences between average values of the mean response time feature was calculated for class 1 and class 2 but not for class 1 and class 3. It could be explained by the fact that the participants had been better acquainted with the application, so they answered questions faster, despite the fact that the third part of the experiment was more difficult. Presumably, there is no statistical significant difference between class 2 and class 3 for mean response time because the second and third part of the experiment included the same number of elements. What is more, a statistically significant difference has been found only for the maximum saccade amplitude feature between class 2 and class 3. This might be explained by the fact that the third part is the most advanced, and then, the participant saccades had the highest amplitude.

## 7. Conclusions

The specific aim of the paper was to conduct a deeper analysis of the features related to the classification of particular cognitive workload levels. It is an important task from the point of view of understanding the influence of cognitive workload level and mental fatigue on the brain cognitive process.

Interpretable machine learning allows to understand which features provide the most valuable information about the examined process. Generally speaking, it is more profitable to understand the reasons behind the decision taken by a machine learning model rather than using it as a black box. Understanding major processes beneath phenomena of interest allows us to build more robust models and perform more effective monitoring of cognitive workload.

For example, in the presented case, due to the interpretable machine learning model, we know which features to focus on and which ones can be neglected, thus allowing lowering the computing cost and obtaining better results. In practice, it is more reasonable to use as few features as possible, since this approach requires less processing power and is more robust.

As a general conclusion, the authors may state that the paper can serve as an example for researchers seeking ideas and techniques to investigate the relationships between mental fatigue and various biomedical measures.

## Figures and Tables

**Table 1:** A review of the literature.

Literature	Number of Participants	Number of Cognitive Workload Levels		Classifier Model	Result	Approach (sd/si)
[35]	34	3	difficulty split	Random forest	0.51 (AUC)	si
			Rash model		0.81 (AUC)	
			stress–strain model		0.67 (AUC)	
[17]	21	3, difficulty split		kNN,	0.332 (F score)	si
[36]	14	2, difficulty split		LDA	0.91 (ACC)	si
[37]	35	2, difficulty split		SVM	0.52 (ACC)	si
[38]	1 (24 recordings)	2, difficulty split		Convolutional deep neural networks	0.95 (ACC)	sd
[39]	1 (24 h of recordings)	2, difficulty split		Convolutional deep neural network	0.934 (ACC)	sd
		3, difficulty split			0.976 (ACC)	
		6, difficulty split			0.945 (ACC)	
[40]	8	2		Deep neural network	0.868 (ACC)	si
[41]	12	7, difficulty split		Artificial Neural Network	0.4–0.98 (ACC)	si
[42]	25	2, difficulty split		Extra Trees	0.768 (ACC)	si
					0.824 (ACC)	sd
		3, difficulty split			0.467 (ACC)	si
					0.637 (ACC)	sd
[43]	37	2, difficulty split		Convolutional deep neural network	0.767 (ACC)	si
					0.861 (AUC)	sd
[44]	16	2, difficulty split		SVM	0.81 (AUC)	si
[45]	92	3, difficulty split		Convolutional deep neural network 3D	0.86 (AUC)	si
[46]	12	2, difficulty split		SVM	0.92 (AUC)	si
[47]	13	4, difficulty split		Deep neural network	0.907 (ACC) 0.896 (F score)	si
[48]	47	2, difficulty split		Forest of Extremely Randomised Trees	0.72 (ACC)	si
[49]	13	4, difficulty split		Deep neural network	0.98 (AUC)	si

**Table 2:** Main classification performance measures obtained for a complete feature set.

Classifier	Recall	Precision	F1 Score	Accuracy	ROC AUC
SVM linear	**0.94 ± 0.05**	**0.95 ± 0.05**	**0.94 ± 0.05**	**0.94 ± 0.05**	**0.99 ± 0.02**
SVM quadratic	0.71 ± 0.10	0.77 ± 0.10	0.71 ± 0.10	0.71 ± 0.10	0.85 ± 0.07
SVM cubic	0.90 ± 0.07	0.92 ± 0.06	0.90 ± 0.07	0.90 ± 0.07	0.98 ± 0.03
Log regression	**0.95 ± 0.05**	**0.96 ± 0.04**	**0.95 ± 0.05**	**0.95 ± 0.05**	**0.99 ± 0.02**
kNN	0.88 ± 0.07	0.90 ± 0.06	0.88 ± 0.07	0.88 ± 0.07	0.96 ± 0.04
Decision Tree	0.89 ± 0.07	0.92 ± 0.05	0.89 ± 0.07	0.89 ± 0.07	0.95 ± 0.04
Random Forest	**0.95 ± 0.05**	**0.96 ± 0.04**	**0.95 ± 0.05**	**0.95 ± 0.05**	**0.99 ± 0.02**
MLP	0.90 ± 0.07	0.92 ± 0.06	0.90 ± 0.07	0.90 ± 0.07	0.98 ± 0.03

The best classification performance results are in bold. The best mean values of separate performance metrics achieved by specific models are presented in the following way: mean ± standard deviation (feature number).

**Table 3:** Main classification performance measures obtained for a selected feature subset.

Classifier	Recall	Precision	F1 Score	Accuracy	ROC AUC
SVM linear	**0.97 ± 0.04 (5)**	**0.97 ± 0.03 (5)**	**0.97 ± 0.04 (5)**	**0.97 ± 0.04 (5)**	**0.99 ± 0.02 (5)**
SVM quadratic	0.92 ± 0.06 (8)	0.93 ± 0.05 (8)	0.92 ± 0.07 (8)	0.92 ± 0.06 (8)	0.98 ± 0.03 (8)
SVM cubic	0.94 ± 0.05 (8)	0.96 ± 0.04 (8)	0.94 ± 0.05 (8)	0.94 ± 0.05 (8)	0.99 ± 0.02 (6)
Log regression	**0.97 ± 0.04 (4)**	**0.97 ± 0.04 (4)**	**0.97 ± 0.04 (4)**	**0.97 ± 0.04 (4)**	**0.99 ± 0.01 (4)**
kNN	0.96 ± 0.05 (8)	0.96 ± 0.04 (8)	0.96 ± 0.05 (8)	0.96 ± 0.05 (8)	0.99 ± 0.02 (5)
Decision Tree	0.90 ± 0.07 (5)	0.92 ± 0.05 (5)	0.90 ± 0.07 (5)	0.90 ± 0.07 (5)	0.95 ± 0.05 (8)
Random Forest	0.95 ± 0.05 (7)	0.96 ± 0.03 (7)	0.95 ± 0.04 (7)	0.95 ± 0.05 (7)	0.99 ± 0.02 (7)
MLP	**0.97 ± 0.05 (5)**	**0.98 ± 0.04 (5)**	**0.97 ± 0.05 (5)**	**0.97 ± 0.05 (5)**	**0.99 ± 0.01 (5)**

Every cell contains a series of numbers, which has to be understood in the following way: mean ± standard deviation (number of features ensuring the best result). The best classification performance results are in bold. The best mean values of separate performance metrics achieved by specific models are presented in the following way: mean ± standard deviation (feature number).

**Table 4:** Separate class feature rankings obtained by interpreting the weights of the LogReg model with elastic net regularization.

No.	Low	Medium	High
1	mean amplitude of saccades	mean response time	response number
2	mean response time	response number	fixation number
3	standard deviation of fixation duration	mean amplitude of saccades	saccade number
4	fixation number	standard deviation of fixation duration	mean amplitude of saccades
5	standard deviation of right pupil diameter	fixation number	mean duration of fixation
6	saccade number	number of errors	maximum saccade amplitude
7	number of errors	mean duration of fixation	mean response time
8	mean of right pupil diameter	saccade number	standard deviation of right pupil diameter
9	mean duration of fixation	mean duration of saccades	mean duration of saccades
10	mean duration of saccades	standard deviation of right pupil diameter	maximum fixation duration
11	mean of blink duration	mean of right pupil diameter	number of errors
12	minimum fixation duration	maximum fixation duration	mean of right pupil diameter
13	response number	maximum saccade amplitude	blink number
14	standard deviation of saccades amplitude	minimum fixation duration	mean of blink duration
15	mean of left pupil diameter	standard deviation of saccades amplitude	standard deviation of left pupil diameter
16	standard deviation of left pupil diameter	mean of left pupil diameter	standard deviation of fixation duration
17	maximum fixation duration	blink number	standard deviation of saccades amplitude
18	maximum saccade amplitude	mean of blink duration	minimum fixation duration
19	blink number	standard deviation of left pupil diameter	mean of left pupil diameter
20	minimum saccade amplitude	minimum saccade amplitude	minimum saccade amplitude

**Table 5:** Mean confusion matrix for SVM classifier with linear kernel.

		True		
		Class 1	Class 2	Class 3
**Predicted**	**Class 1**	5.62	0.38	0
	**Class 2**	0.23	5.77	0
	**Class 3**	0	0.005	5.995

**Table 6:** Mean confusion matrix for Logistic Regression classifier with linear kernel.

		True		
		Class 1	Class 2	Class 3
**Predicted**	**Class 1**	5.64	0.36	0
	**Class 2**	0.22	5.78	0
	**Class 3**	0	0.005	5.995

**Table 7:** Mean confusion matrix for MLP.

		True		
		Class 1	Class 2	Class 3
**Predicted**	**Class 1**	5.64	0.36	0
	**Class 2**	0.16	5.84	0
	**Class 3**	0	0	6

**Table 8:** The results of one-way ANOVA analysis.

	ANOVA	Post-hoc Test		
Features	*p*-Value	*p*-Value Class 1–Class 2	*p*-Value Class 1–Class 3	*p*-Value Class 2–Class 3
mean response time	<0.001	<0.001	<0.001	0.069
standard deviation of fixation duration	0.002	0.003	0.008	0.947
maximum fixation duration	0.009	0.011	0.04	0.877
maximum saccade amplitude	0.002	0.411	0.001	0.046
mean saccade amplitude	<0.001	<0.001	<0.001	0.088
standard deviation of left pupil diameter	0.006	0.016	0.011	0.993
standard deviation of right pupil diameter	0.023	0.069	0.029	0.934

## Data Availability

The data presented in this study are available on request from the corresponding author.
